# Census Bulletin No. 11

**Published:** 1890-11

**Authors:** 


					﻿Census Bulletin No. 11
Shows the statistics of transportation in the United States, including i*apid
transit in the cities.
				

